# ΔNp63 intronic miR-944 is implicated in the ΔNp63-mediated induction of epidermal differentiation

**DOI:** 10.1093/nar/gkv735

**Published:** 2015-07-21

**Authors:** Kyu-Han Kim, Eun-Gyung Cho, Seok Jong Yu, Hyojin Kang, Yoon-Jin Kim, Sang Hoon Kim, Tae Ryong Lee

**Affiliations:** 1 Bioscience Research Division, R&D Unit, AmorePacific Corporation, Yongin-si, Gyeonggi-do 446-729, Republic of Koreaf; 2 National Institute of Supercomputing and Networking, Korea Institute of Science and Technology Information, Daejeon 305-806, Republic of Korea; 3 Department of Biology, Kyung Hee University, Seoul 130-701, Republic of Korea

## Abstract

ΔNp63 is required for both the proliferation and differentiation of keratinocytes, but its role in the differentiation of these cells is poorly understood. The corresponding gene, *TP63*, harbors the *MIR944* sequence within its intron. However, the mechanism of biogenesis and the function of miR-944 are unknown. We found that miR-944 is highly expressed in keratinocytes, in a manner that is concordant with that of *ΔNp63* mRNA, but the regulation of miR-944 expression under various conditions did not correspond with that of *ΔNp63*. Bioinformatics analysis and functional studies demonstrated that *MIR944* has its own promoter. We demonstrate here that *MIR944* is a target of ΔNp63. Promoter analysis revealed that the activity of the *MIR944* promoter was markedly enhanced by the binding of ΔNp63, which was maintained by the supportive action of AP-2 during keratinocyte differentiation. Our results indicated that miR-944 biogenesis is dependent on ΔNp63 protein, even though it is generated from *ΔNp63* mRNA-independent transcripts. We also demonstrated that miR-944 induces keratin 1 and keratin 10 expression by inhibiting ERK signaling and upregulating p53 expression. Our findings suggested that miR-944, as an intronic miRNA and a direct target of ΔNp63, contributes to the function of ΔNp63 in the induction of epidermal differentiation.

## INTRODUCTION

The protein p63, which belongs to the p53/p63/p73 family of transcription factors, plays an important role in normal epidermal stratification, the maintenance of pluristratified epithelia, and the proliferative potential of epithelial stem cells (1,2). This protein exists as tissue-specific isoforms, transcribed from alternative promoters, which give rise to TAp63 containing the transactivation (TA) domain and an N-terminal truncated isoform (ΔNp63) lacking the TA domain. Alternative splicing at the 3′-end also generates three splicing isoforms (α, β and γ) (2). In the epidermis, expression of ΔNp63 is restricted to the proliferative basal epidermal cells and plays a role in the maintenance of the proliferative capability of cells, and its expression is decreased in differentiated layers (3,4). However, it is known that ΔNp63 also plays a critical role in the commitment to epidermal differentiation. The depletion of ΔNp63 results in defective expression of early differentiation markers (5,6). The molecular mechanism by which ΔNp63 governs epidermal proliferation has been relatively well studied (5,7); however, it is not clear how ΔNp63 could induce epidermal differentiation.

MicroRNAs (miRNAs) are small non-coding endogenous RNAs of approximately 21–23 nucleotides. Genomic sequences that encode miRNAs are spread widely throughout the genome (8,9). Depending on their location in the genome, miRNAs can be classified as either intragenic miRNAs or intergenic miRNAs. Most intragenic miRNAs are located in the introns of host genes and are called intronic miRNAs; these account for approximately half of the known vertebrate miRNAs.

It has long been believed that intronic miRNAs are generated from their host transcription units and should thus be coordinately expressed with their respective host gene mRNA. However, patterns of discordant expression between host genes and their embedded intronic miRNAs have been reported (10–14). Moreover, genomic analysis revealed that approximately one-third of intronic miRNAs have their own transcription initiation regions, including promoters, transcription start sites, and conserved transcription factor-binding sites (15,16). In addition, it has also been reported that alternative splicing and alternative polyadenylation are involved in the biogenesis of some intronic miRNAs (17,18) and that mirtrons, which reside in short introns, are also generated through their own unique process (19).

Furthermore, many studies have reported that intronic miRNAs that are co-expressed with their host genes play important roles in the regulation of host gene expression or function (20–22). However, it is not clear whether intronic miRNAs that are expressed host gene-independently have such an influence on their host genes. Therefore, a detailed investigation of the genetic relationship between individual intronic miRNAs and their host genes is required for a complete understanding of the function and biology of intronic miRNAs.

Intron 4 of human *TP63* contains the gene for miR-944 (*MIR944*). Although the function of p63 has been extensively investigated, no studies on the generation of miR-944 and its relationship to its host gene and its function have been reported. In this study, we showed that, although miR-944 is generated from *TP63*-independent transcriptional units, *MIR944* transcription is dependent on the transactivating role of ΔNp63 within the promoter region of *MIR944*. The findings of this study extend the current view on the regulatory mechanisms of intronic miRNAs. Additionally, our results demonstrated that miR-944 facilitates the ΔNp63-mediated induction of epidermal differentiation by downregulating ERK signaling and upregulating p53 expression.

## MATERIALS AND METHODS

### Cell culture and treatment

Human primary keratinocytes were obtained from Lonza (Walkersville, MD, USA) and were maintained in serum-free KGM-Gold™ media with Bullet-kit supplements (Lonza). Human epithelial transformed HaCaT cells were obtained from Cell Lines Service (CLS, Eppelheim, Germany) and were maintained in Dulbecco's modified Eagle's medium (DMEM) with 1% penicillin/streptomycin and 10% heat-inactivated fetal bovine serum (FBS, Gibco, Carlsbad, CA, USA). Human epithelial carcinoma A431 cells, human placenta choriocarcinoma JAR cells, and human prostate carcinoma LNCaP, DU145 and PC3 cells were obtained from the Korean Cell Line Bank (KCLB, Seoul, Korea) and maintained in Roswell Park Memorial Institute medium with 1% penicillin/streptomycin and 10% heat-inactivated FBS. Human primary dermal fibroblasts from neonatal foreskin were obtained from Lonza and were maintained in DMEM with 1% penicillin/streptomycin and 10% heat-inactivated FBS. Human primary dermal melanocytes from neonatal foreskin were purchased from Cascade Biologics (Portland, OR, USA) and were cultured in Medium 254 (Cascade Biologics) with Human Melanocyte Growth Supplement (Cascade Biologics). Human melanoma WM115 and WM266-4 cells were obtained from the American Type Culture Collection (Manassas, VA, USA), and human melanoma SK-MEL1, SK-MEL-2, SK-MEL-3 and SK-MEL-5 cells were obtained from the KCLB and were maintained in Eagle's minimal essential medium (ATCC) with 1% penicillin/streptomycin and 10% heat-inactivated FBS. Human pigmented melanoma MNT1 cells were maintained in MNT1 culture medium, consisting of MEM supplemented with 10% DMEM, 1% penicillin/streptomycin, 20% heat-inactivated FBS, and 10 mM HEPES. Human placenta choriocarcinoma JEG3 and RD cells and human cervical carcinoma HeLa cells were obtained from the KCLB and maintained in DMEM containing 1% penicillin/streptomycin and 10% heat-inactivated FBS. All cells were grown at 37°C under 5% CO_2_.

To examine the effects of epigenetic reagents, HeLa cells were treated with 5 μM 2′-deoxy-5-azacytidine (5-aza, Cat. #A3656, Sigma, St Louis, MO, USA) for 3 days. After treatment, 1 μM trichostatin A (TSA, Cat. #T1952, Sigma) was added directly to the medium, followed by 1 day of further incubation.

To examine the effect of ERK signaling, keratinocytes were treated with 5 μM PD98059 (Cell Signaling Inc.) for 2 days.

### RNA extraction

Total RNA, including small RNAs, was extracted using either TRIzol reagent (Invitrogen, Carlsbad, CA, USA) or the mirVANA miRNA isolation kit (Ambion, Austin, TX, USA) as per the manufacturers’ protocols.

To determine whether miR-944 is incorporated into the RNA-induced silencing complex (RISC), we performed an immunoprecipitation experiment in human primary keratinocytes. Cells were lysed and subjected to AGO2-immunoprecipitation (AGO2-IP) using the Human AGO2 microRNA isolation kit (Wako Pure Chemical, Osaka, Japan) according to the manufacturer's instructions. As a negative control, immunoprecipitation was also performed using IgG beads prepared with an antibody immobilization bead kit (Wako Pure Chemical). In addition, we also used WM266-4 melanoma cells as a negative control for the detection of miR-944. After elution, extracted RNAs were reverse-transcribed and subjected to a TaqMan miRNA assay (Applied Biosystems, Foster City, CA, USA).

### Reverse transcription quantitative PCR (RT-qPCR)

The total RNA from each sample was reverse transcribed to its corresponding cDNA using a Superscript Reverse Transcriptase II kit (Invitrogen). Real-time analysis of mRNA expression was performed using TaqMan Universal Master PCR mix (Applied Biosystems). Gene expression was quantified using the comparative ΔΔCT method. cDNA samples were analyzed using the probes listed in Supplementary Table S1. Cycle threshold values of the mRNAs were normalized against levels of endogenous *RPLP0* (Applied Biosystems), as specifically recommended for keratinocytes. To examine the expression of mature miRNAs, total RNA was reverse-transcribed using the TaqMan MicroRNA Reverse Transcription kit (Applied Biosystems) according to the manufacturer's instructions and were then subjected to a TaqMan miRNA assay (Applied Biosystems) using the respective probes listed in Supplementary Table S1. *RNU48* snRNA was used for the normalization of miRNA expression. RT-qPCR analysis was performed on an ABI7500FAST (Applied Biosystems).

### Transfection

To identify the factors affecting miR-944 expression, human primary keratinocytes were transfected with the siRNAs (Bioneer Co., Daejeon, Korea) listed in Supplementary Table S2, using RNAiMAX lipofectamine reagent (Invitrogen) according to the manufacturer's instructions.

To study the function of miR-944, human primary keratinocytes were transfected with mimics (Cat. #4464066) or inhibitors (Cat. #4464084) of miR-944 (Ambion) using RNAiMAX lipofectamine reagent (Invitrogen) according to the manufacturer's instructions.

### *In situ* hybridization (ISH) detection of miRNA

ISH was performed on paraffin-embedded sections of normal skin tissue obtained from Zyagen (San Diego, CA, USA) and psoriasis skin tissue obtained from Creative Bioarray (Shirley, NY, USA). Skin tissue slides were incubated with locked nucleic acid (LNA) probes (5′-digoxin [DIG]- and 3′-DIG-labeled LNA probes specific for miR-944 or scrambled probes with no homology to known vertebrate miRNAs; Exiqon, Woburn, MA, USA) overnight in a hybridization chamber at 40°C. After washing, probe binding was detected by incubating the slides with a sheep anti-DIG-alkaline phosphatase antibody (Roche, Mannheim, Germany). Slides were visualized after incubation in nitro blue tetrazolium chloride/5-bromo-4-chloro-3-indolyl phosphate (NBT/BCIP) substrate solution (Thermo Scientific, Rockford, IL, USA).

### 5′ Rapid amplification of cDNA end analysis (5′RACE)

Poly A+ RNAs from human primary keratinocytes were extracted using the Oligotex Direct mRNA Mini kit (Qiagen, Valencia, CA, USA) as per the manufacturer's protocol. Next, the 5′**-**end of primary *MIR944* transcripts was determined by 5′RACE using a SMARTer RACE cDNA Amplification kit (Clontech, Mountain View, CA, USA), followed by a nested PCR reaction. The first-stage of PCR utilized Universal Primer mix (forward primer) and a primary *MIR944* transcript-specific primer (reverse primer: 5′-GGGCCTTTATTTGTCTTCCCTGCCA-3′). The second-stage PCR was performed with the Nested Universal Primer A (forward primer) and another primary *MIR944* transcript-specific primer (reverse primer: 5′-GAGAGGCTGCAGGGAAGAGCAATCT-3′). The nested RACE products were separated on an agarose gel, extracted, cloned into the pMD18-T vector (Takara Bio Inc., Otsu, Japan), and sequenced.

### Construction of a luciferase reporter and expression vectors

The *ΔNp63* and *MIR944* promoter regions were amplified from human genomic DNA by PCR using the primer sets listed in Supplementary Table S3. PCR products were cloned into the pGL3-Basic vector (Promega, Madison, WI, USA). The mutant constructs were generated using the QuikChange Site-Directed Mutagenesis kit (Stratagene, La Jolla, CA, USA), according to the manufacturer's instructions, using the primer sets listed in Supplementary Table S3. Human primary keratinocytes or WM266-4 melanoma cells were transfected with each of the constructed luciferase constructs. All cells were cotransfected with the β-galactosidase expression vector (pSV-β-galactosidase). *ΔNp63* expression vectors were purchased from Addgene (Cambridge, MA, USA). TFAP2A and TFAP2C expression vectors were constructed with the primers listed in Supplementary Table S3.

Partial segments of the 3′ UTR of *MAPK1, FGF2, NRAS, RRAS2* mRNA containing the predicted miR-944-binding sequences were PCR-amplified using the primers listed in Supplementary Table S3. The PCR products were cloned into the pMIR-REPORT Firefly Luciferase reporter vector (Ambion, Austin, TX, USA). Site-directed mutagenesis was performed by overlapping PCR the primers listed in Supplementary Table S3.

### Luciferase assay

Luciferase and β-galactosidase activities were measured using a Luciferase Assay kit (Promega) and a β-Galactosidase Enzyme Assay System (Promega) according to the manufacturer's protocol.

### Chromatin immunoprecipitation (ChIP)-PCR

ChIP was performed using a ChIP High-Sensitivity kit (Active Motif, Carlsbad, CA, USA) according to the manufacturer's instructions. Fixed cells were lysed and then sonicated to shear the chromatin (Vibra-Cell, Sonics & Materials, Newtown, CT, USA). Immunoprecipitates were incubated overnight at 4°C with the following antibodies: IgG antibody (Millipore, Billerica, MA, USA), anti-RNA-Pol antibody (Millipore), anti-ΔNp63 antibody (N-16 clone; Santa Cruz Biotechnology, Santa Cruz, CA, USA), anti-TFAP2A antibody (3B5 clone; Santa Cruz Biotechnology), anti-TFAP2C antibody (H-77 Clone; Santa Cruz Biotechnology), anti-histone H3 (acetyl-K9) antibody (Ambion), anti-histone H3 (acetyl-K27) antibody (Ambion), and anti-histone (tri-methyl-K4) antibody (Millipore). After washing, DNA was eluted from the beads, and cross-links were reversed. Quantitative PCR was performed on the ChIP-enriched DNA using the TaqMan Universal Master PCR mix (Applied Biosystems). Primers are listed in Supplementary Table S1.

### Microarray

Keratinocytes were transfected with a miR-944 mimic or a negative-control mimic (NC mimic). The mRNA expression profile regulated by miR-944 overexpression was generated using an Affymetrix GeneChip Human Genome U133 Plus 2.0 Array (Affymetrix, Santa Clara, CA, USA).

### Western blotting

Cells were lysed with a lysis buffer (20 mM Tris [pH 7.5], 0.1% Triton X-100, 0.5% deoxycholate, 1 mM PMSF, 10 μg/ml aprotinin, and 10 μg/ml leupeptin) and then cleared via centrifugation at 4°C. The total protein concentration was determined using a Bio-Rad Protein Assay kit (Bio-Rad, Hercules, CA, USA) according to the manufacturer's instructions, and the obtained proteins were subjected to sodium dodecyl sulfate-polyacrylamide gel electrophoresis. After transferring the proteins onto nitrocellulose membranes and blocking the membranes, the membranes were incubated overnight at 4°C with antibodies in phosphate-buffered saline containing 0.1% Tween 20. The primary antibodies employed for immunoblotting were as follows: anti-β-actin antibody (Santa Cruz Biotechnology), anti-KRT1 antibody (Abcam), anti-KRT10 antibody (Covance, Richmond, VA, USA), anti-p53 antibody (Wako), p44/42 ERK antibody (Cell Signaling Inc., Beverly, MA, USA), phospho-p44/42 ERK antibody (Cell Signaling Inc.), U170K antibody (Santa Cruz Biotechnology), U2AF65 (Santa Cruz Biotechnology), PRP8 antibody (Santa Cruz Biotechnology), FGF2 antibody (Santa Cruz Biotechnology), and NRAS antibody (Santa Cruz Biotechnology). All protein bands were detected using an ECL system (Amersham Pharmacia Biotech, Piscataway, NJ, USA).

### Bioinformatics analysis

To analyze *TP63* in normal and psoriatic skin, the Affymetrix GeneChip Human Genome U133 Plus Array data set GSE14905 was downloaded from NCBI GEO (http://www.ncbi.nlm.nih.gov/geo/query/acc.cgi?acc=GSE14905) and analyzed with R statistical software. To analyze miR-944 expression in normal and psoriatic skin, the Illumina GAIIx platform data set GSE31037 was downloaded from NCBI GEO (http://www.ncbi.nlm.nih.gov/geo/query/acc.cgi?acc=GSE31037) and analyzed with the miRanalyzer default algorithm (http://bioinfo5.ugr.es/miRanalyzer/miRanalyzer.php). One-way ANOVA was performed to determine if the gene was differentially expressed between normal and psoriatic skin.

To assess binding of ΔNp63 to the *MIR944* promoter in keratinocytes, the Illumina Genome Analyzer II platform data set GSE32061 was downloaded from NCBI GEO (http://www.ncbi.nlm.nih.gov/geo/query/acc.cgi?acc=GSE32061) and analyzed. The p63-binding region of the *MIR944* promoter region was visualized using the integrated genome viewer.

## RESULTS

### Identification of the *TP63* intronic miR-944 in keratinocytes

**Figure 1.**
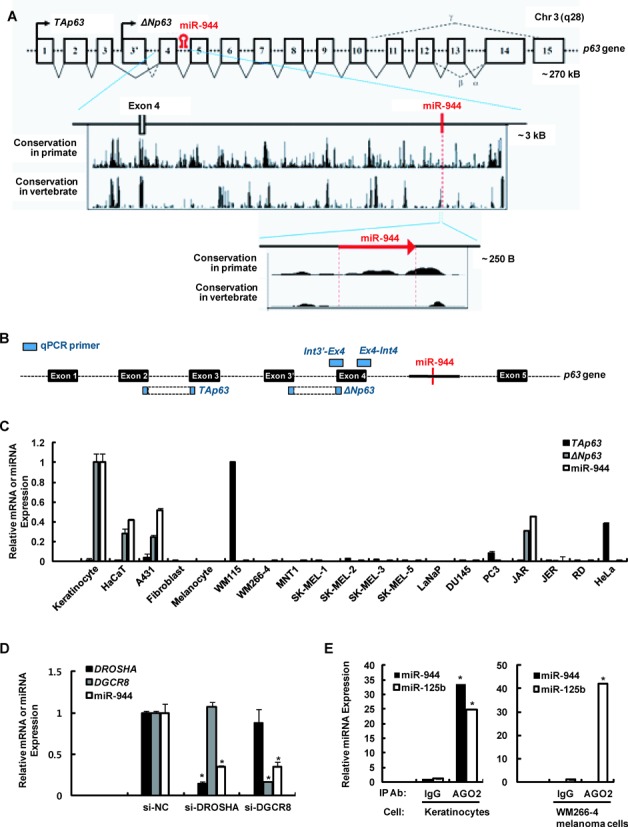
The primate-specific microRNA miR-944 is highly expressed in keratinocytes. (**A**) Schematic representation of human *TP63* and *MIR944*. *TP63* exon numbers are noted. Alternative promoters (P1 and P2) give rise to the *TAp63* and *ΔNp63* transcript isoforms, respectively. *MIR944* (red hairpin) resides within intron 4 of human *TP63*. Analysis using the University of California, Santa Cruz (UCSC) Genome Brower demonstrates the evolutionary conservation of the *TP63* genomic fragments harboring exon 4 and *MIR944* in primates and vertebrates. (**B**) The positions of the qPCR primers used in this study are indicated on human *TP63*. ‘*TAp63’*, ‘*ΔNp63’*, and ‘*p63’* probes can detect exons 2 and 3, 3′ and 4, and 4 and 5, respectively. The ‘*Int3-Ex4’* and ‘*Ex4-Int4*’ probes can detect the intron 3′–exon 4 junction region and the exon 4–intron 4 junction region, respectively. (**C**) Expression levels of *TAp63* mRNA, *ΔNp63* mRNA, and miR-944 in various human cell lines were analyzed using RT-qPCR. *TAp63* mRNA and *ΔNp63* mRNA expression levels were normalized to peptidylprolyl isomerase A (*PPIA*) mRNA expression, and miR-944 expression was normalized to the expression of the *RNU48* small RNA. Data represents the mean ± SD of triplicate biological samples. The values of *ΔNp63*, the relative expression of miR-944 obtained from keratinocytes, and the relative expression of *TAp63* obtained from WM115 cells were all set as 1. (**D**) Human primary keratinocytes were transfected with 50 nM siRNA against *DROSHA* (si-DROSHA), *DGCR8* (si-DGCR8), or a negative control siRNA (si-NC). After 2 days, RNA was extracted, and expression levels were analyzed using RT-qPCR. *DROSHA* and *DGCR8* mRNA levels were normalized to the expression levels of ribosomal protein large P0 (*RPLP0*) mRNA, and miR-944 expression was normalized to *RNU48* small RNA levels. Data represent the means ± SD of triplicate biological samples and are representative of three different experiments. *P < 0.05 versus si-NC, unpaired Student's *t*-test. (**E**) Human primary keratinocytes and WM266-4 melanoma cells were immunoprecipitated with the AGO2 or IgG antibody. RNA was extracted from immunoprecipitates, and miR-944 expression was analyzed. As a positive control, miR-125b expression was also examined. Data represent the means ± SD of triplicate biological samples and are representative of three different experiments. *P < 0.05 versus IgG, unpaired Student's *t*-test.

The stem–loop forming precursor of miR-944 resides in intron 4 of *TP63*, on chromosome 3q28 (Figure 1A). Conservation analyses using PhyloP in the University of California, Santa Cruz (UCSC) Genome Browser and phylogenetic trees indicated that the nucleotide sequences of the mature miR-944 and the *MIR944* stem-loop are only conserved within primates, while the homologues of the *TAp63* and *ΔNp63* mRNAs are more widely conserved in vertebrates, suggesting that *MIR944* is a relatively young gene in the evolutionary hierarchy (Figure 1A and Supplementary Figure S1).

We further investigated in which cells miR-944 and the host gene *TP63* are expressed. To assay the expression of each of the various *TP63* isoforms, we designed several primers for TaqMan-based real-time quantitative PCR (RT-qPCR) experiments, as shown Figure 1B. We then examined the expression patterns of these genes in several human primary cutaneous cells and several classical cell lines that had been derived from the skin, prostate, and placenta. Of the cells derived from skin tissue, primary keratinocytes and keratinocyte-derived cell lines (HaCaT and A431) all highly expressed *ΔNp63* mRNA and mature miR-944, but did not express *TAp63* mRNA (Figure 1C). The JAR cell line, derived from placental choriocarcinoma, showed the same expression pattern as keratinocytes.

Mature miRNAs are generated from long primary transcripts containing miRNA sequences through cropping and dicing processes. The cropping process is mediated by the microprocessor, a protein complex containing DROSHA and DGCR8, and generates intermediate molecules (pre-miRNAs) (8). These intermediates are subsequently cleaved by DICER into miRNA duplexes, and mature miRNAs direct the RNA-induced silencing complex (RISC) to the 3′untranslated region (3′UTR) of their target mRNA (23). To determine whether miR-944 is processed by this general pathway, we first transfected human primary keratinocytes with siRNA against *DROSHA* or *DGCR8*, and performed RT-qPCR after 2 days of incubation. As shown in Figure 1D, the expression levels of miR-944 were decreased in *DROSHA*- and *DGCR8*-depleted keratinocytes, implying that miR-944 is generated through the action of the microprocessor complex.

Next, we did immunoprecipitation experiments using an antibody against AGO2, an essential component of RISC, in keratinocytes, which express miR-944, and in WM-266-4 melanoma cells, which do not express miR-944, and subsequently performed RT-qPCR. As a positive control, we also examined miR-125b expression in both cell types (24,25). RT-qPCR revealed that miR-944 is efficiently loaded onto the RISC complex in keratinocytes (Figure 1E). Thus, we concluded that miR-944 is a primate-specific miRNA that is generated by the microprocessor complex; it functions in an AGO2-dependent manner, and is highly expressed in keratinocyte-derived cells in accordance with *ΔNp63* mRNA expression.

### Independent regulation of miR-944 and its host gene *ΔNp63* expression

**Figure 2.**
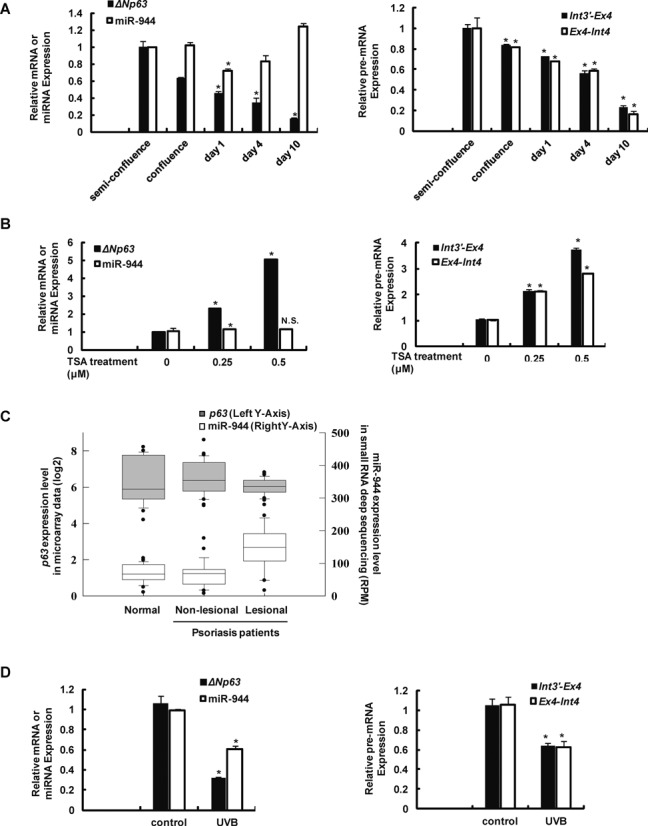
Discordant regulation of *ΔNp63* and miR-944 expression under various conditions. (**A**) Human primary keratinocytes were differentiated by cultivating for the indicated time period after the replacement of culture medium with a medium containing a high calcium concentration (1.2 mM). The relative expression levels of *ΔNp63* and miR-944 (left panel) and the expression of precursor *TP63* mRNA (right panel) were examined during calcium-induced keratinocyte differentiation. Data represent the means ± SD of triplicate biological samples and are representative of four different experiments. *P < 0.05 versus semi-confluence, unpaired Student's *t*-test. (**B**) Human primary keratinocytes were treated with the indicated concentration of TSA for 4 days. The relative expression levels of *ΔNp63* and miR-944 (left panel) and the expression of precursor *TP63* mRNA (right panel) were examined. Data represent the means ± SD of triplicate biological samples and are representative of three different experiments. *P < 0.05 versus TSA 0 μM, unpaired Student's *t*-test. (**C**) Box plots showing normalized gene expression levels of *TP63* and *MIR944* in the skin of healthy individuals and in the lesional and non-lesional skin of psoriasis patients, which were examined using GEO deposited data (*TP63* expression, GEO accession number: GSE14905; miR-944 expression, GEO accession number: 31037). (**D**) Human primary keratinocytes were exposed to 30 mJ/cm^2^ of UVB daily for 3 consecutive days. The relative expression levels of *ΔNp63* and miR-944 (left panel) and the expression of precursor *TP63* mRNA (right panel) were examined. Data represent the means ± SD of triplicate biological samples and are representative of three different experiments. *P < 0.05 versus control, unpaired Student's *t*-test.

Next, we examined how miR-944 expression is regulated in keratinocytes during differentiation. We stimulated differentiation of keratinocytes by cultivating them in a medium containing a high calcium concentration after the cells had reached 100% confluence. *ΔNp63* mRNA expression gradually decreased during the course of differentiation, as has been well documented previously (4). Strangely, however, unlike *ΔNp63* mRNA, miR-944 did not show any significant changes in expression during the differentiation process (left panel; Figure 2A). The steady expression of miR-944 during epidermal differentiation was also confirmed by *in situ* hybridization (ISH) experiments on skin sections, using miR-944-specific LNA probes. In normal skin tissue, miR-944 is widely detected in almost all layers of the epidermis (Supplementary Figure S2), whereas it is well-known that mRNA and protein expression of ΔNp63 is restricted to the basal and lower spinous layers of the epidermis (1).

We observed several instances of discordant expression of miR-944 and *ΔNp63* mRNA, in addition to the differences in the expression during keratinocyte differentiation. First, the response to epigenetic modifying reagents differed. It had previously been reported that the treatment of keratinocytes with trichostatin A (TSA), a histone deacetylase (HDAC) inhibitor, increased the expression of *ΔNp63* mRNA (26). Similarly, we observed that *ΔNp63* mRNA expression was significantly increased by TSA treatment, in a concentration-dependent manner. However, miR-944 expression remained unchanged, as shown in Figure 2B. In addition, the differential induction of *ΔNp63* mRNA and miR-944 by TSA was also observed in HeLa cells. We first treated HeLa cells, which do not express either of these genes, with the DNA methylation inhibitor 2′-deoxy-5-azacytidine (5′aza) to induce the expression of both genes and then added TSA. Interestingly, TSA treatment blocked the induction of *ΔNp63* mRNA expression by 5′aza, but further increased miR-944 expression in HeLa cells (Supplementary Figure S3). These results strongly suggested that the expression of miR-944 and *ΔNp63* mRNA are differently regulated by TSA.

We also revealed discrepant expression of miR-944 and *ΔNp63* in psoriasis, a common, chronic human skin disorder. Pathogenic pathways and molecular components of this disease have been extensively examined using gene expression profiling. We analyzed the expression of miR-944 in deep sequencing data of small RNAs, which were obtained from an open-source database (27). Interestingly, we found that the read count of mature miR-944 was considerably increased in psoriatic lesions (Figure 2C and Supplementary Figure S4). However, it has previously been reported that *ΔNp63* mRNA expression is slightly reduced in such lesions (28). Therefore, we concluded that miR-944 expression correlates poorly with that of *ΔNp63* in psoriasis. Unlike the above cases, ultraviolet B (UVB) irradiation induced decreased expression of both *ΔNp63* mRNA and miR-944 (Figure 2D).

### Host gene-independent *MIR944* promoter

Differences in the stability of mature mRNA and miRNA could possibly explain the above discrepancies, as it is generally thought that miRNAs are relatively more stable than mRNAs (29). However, this possibility was excluded, because our previous results showed that expression of mature miR-944 was significantly decreased within 2 days after transfection of cells with *DROSHA* siRNA (Figure 1D). In addition, we also observed that the reduction of mature miR-944 expression was greater relative to that of other mature miRNAs (Supplementary Figure S5). Thus, the stability of miR-944 is not the critical factor in this apparent dichotomy.

As a second explanation, it is possible that transcriptional regulation of the precursor mRNA (pre-mRNA) plays a critical role in miR-944 processing. For example, in the case of miR-198/FSTL1, the binding of the RNA-binding protein to the primary transcript determines the context of miRNA/mRNA expression (30); the expression of a single pre-mRNA, which comprises both the primary miRNA transcript and the mRNA, persists even when miRNA/mRNA expression patterns change. However, in our study, the expression pattern of *ΔNp63* pre-mRNA was similar to that of *ΔNp63* mRNA (right panels; Figure 2A, B and D), unlike miR-198/FSTL1. These results indicated that *ΔNp63* pre-mRNA may not function as the primary miRNA transcript of miR-944.

Thirdly, splicing activity could affect the biogenesis of intronic miRNA from pre-mRNA (31). Therefore, we investigated whether miR-944 biogenesis is dependent on splicing activity. However, knockdown of three different core components of the spliceosome, U170K, U2AF65 and PRP8 with the respective siRNAs had no significant effect on miR-944 expression (Supplementary Figure S6), ruling out this possibility.

**Figure 3.**
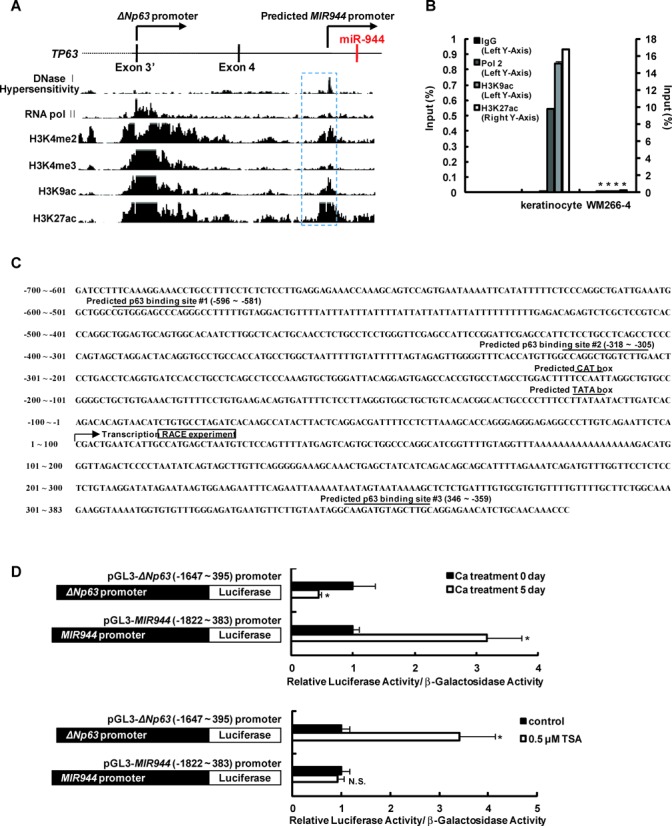
Identification of a *MIR944* promoter that is independent of the host gene *ΔNp63* promoter. (**A**) UCSC Genome Browser view of DNase I hypersensitivity, RNA pol II, H3K4me2, H3K4me3, H3K9ac and H3K27ac tracks obtained for normal human epidermal keratinocytes at *TP63*. DNase I hypersensitivity marks transcriptionally active regions; H3K4me2 marks promoter and enhancer activity; H3K4me3 marks promoter activity; and H3K9Ac and H3K27Ac mark open chromatin structure and transcriptional initiation. The predicted promoter of *MIR944* is shown in the blue box (dashed line). (**B**) ChIP-qPCR results for RNA pol II, H3K9ac and H3K27Ac binding to the region of the *MIR944* promoter in both keratinocytes and WM266-4 melanoma cells. Data represent the means ± SD of triplicate biological samples and are representative of three different experiments. *P < 0.05 versus keratinocyte, unpaired Student's *t*-test. (**C**) Nucleotide sequences in the *MIR944* promoter region (−700 to +383). Areas marked with a line indicate possible CAT and TATA boxes and predicted p63-binding sites. The RACE clone closely matches the predicted transcript on the UCSC Genome Browser. The first nucleotide of the transcript from the RACE experiment is numbered as 1. (**D**) Human primary keratinocytes were transfected with *ΔNp63* promoter-luciferase (pGL3-*ΔNp63* [−1647 to +395] promoter) or a *MIR944* promoter-luciferase construct (pGL3-*MIR944* [−1822 to 383] promoter). (Upper panel) Transfected keratinocytes were differentiated by cultivating the cells for the indicated time period in a high-calcium medium (1.2 mM) for 5 days, and luciferase and β-galactosidase activities were then measured. (Bottom panel) Transfected keratinocytes were treated with 0.5 μM TSA. After 1 day of incubation, luciferase and β-galactosidase activities were measured. Luciferase activities were normalized to β-galactosidase activities. Data represent the means ± SD of triplicate biological samples and are representative of three different experiments. *P < 0.05 versus Ca treatment 0 day or control, unpaired Student's *t*-test.

Finally, we hypothesized that miR-944 could be transcribed and regulated as an independent transcription unit through its own transcriptional regulatory elements. To test this possibility, we first analyzed chromatin signatures that have been established as markers of transcriptionally active promoters in the genomic region of *MIR944*. Dimethylation and trimethylation of Lys 4 of histone 3 (H3K4me2 and H3K4me3) and acetylation of Lys 9/27 of histone 3 (H3K9ac and H3K27ac) mark promoters, and DNase I hypersensitive sites are chromatin regions that have lost their condensed structures and are transcriptionally active. In order to predict a *MIR944* promoter sequence, we used chromatin immunoprecipitation-sequencing (ChIP-Seq) data from human keratinocytes deposited in the UCSC Genome Browser. This analysis allowed us to predict the transcription initiation region, which is located approximately 5 kb downstream of the genomic sequence containing *MIR944* (Figure 3A). To confirm this, we compared the chromatin status of keratinocytes in the predicted promoter of *MIR944* with that of WM266-4 melanoma cells, which do not express miR-944. The ChIP-qPCR data showed that the predicted transcription initiation occurred in keratinocytes, but not in WM266-4 melanoma cells (Figure 3B).

Moreover, analysis of chromatin state segmentation also predicted a promoter for the independent transcription of *MIR944* (Supplementary Figure S7). We then performed 5′-RACE and determined that the transcription start site is located near the region that was predicted by the genomic analysis (Figure 3C).

Next, to confirm the functionality of the predicted promoter, we constructed promoter-luciferase vectors containing approximately 2 kb of sequence encompassing either the *ΔNp63* or the putative *MIR944* promoter (*ΔNp63* promoter-luciferase and *MIR944* promoter-luciferase, respectively), as illustrated in Figure 3D. Keratinocytes were transfected with either of these vectors, and differentiation was induced. The activity of the *MIR944* promoter was upregulated, whereas that of the *ΔNp63* promoter was suppressed (upper panel; Figure 3D). Moreover, TSA treatment increased the activity of the *ΔNp63* promoter, but not that of the *MIR944* promoter (bottom panel, Figure 3D). These results agree with the expression data of the individual RNAs, which are shown in Figure 2A and B.

### *MIR944* is a target of ΔNp63

**Figure 4.**
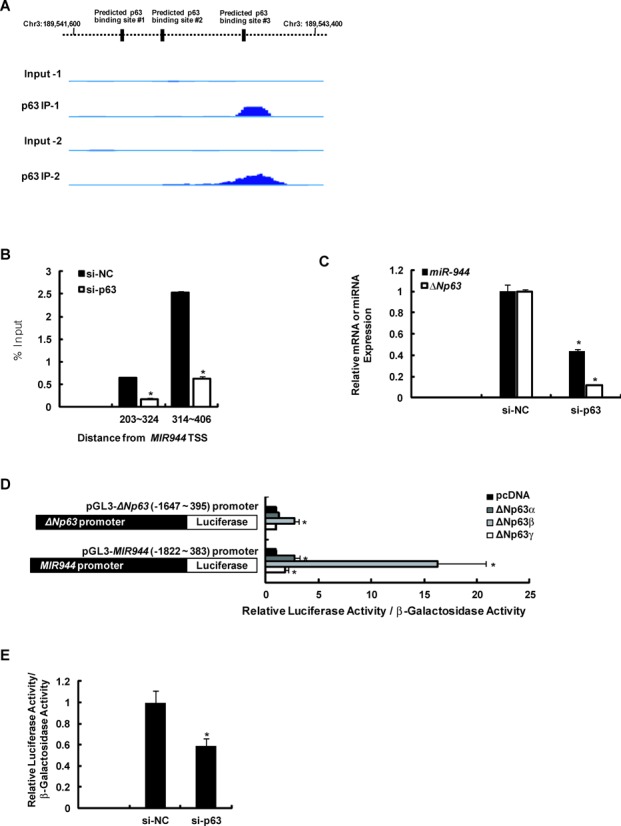

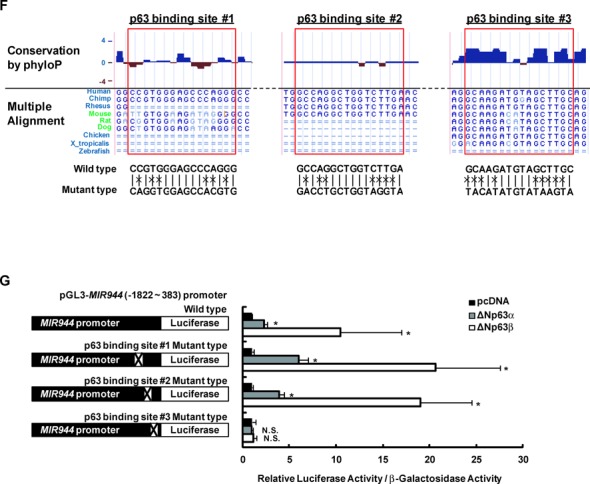
ΔNp63 is responsible for the activation of the *MIR944* promoter. (**A**) ChIP-seq results obtained from GEO (accession number GSE32061) for the *MIR944* promoter for two independent biological replicates. (**B**) ChIP-qPCR results for ΔNp63 binding to the *MIR944* promoter in keratinocytes that had been transfected with the si-NC compared to those transfected with *TP63* siRNA (si-p63). Data represent the means ± SD of triplicate biological samples and are representative of three different experiments. *P < 0.05 versus si-NC, unpaired Student's *t*-test. (**C**) Reduction in miR-944 expression with p63 depletion in keratinocytes. Keratinocytes were transfected with 50 nM siRNA against *TP63* mRNA (si-p63) or si-NC. After 2 days, RNA was extracted, and expression levels were analyzed using RT-qPCR. The levels of *ΔNp63* mRNA were normalized to the expression of *RPLP0*, and those of miR-944 were normalized to levels of *RNU48* small RNA. Data represent the means ± SD of triplicate biological samples and are representative of three different experiments. *P < 0.05 versus si-NC, unpaired Student's *t*-test. (**D**) WM266-4 melanoma cells, which do not express *ΔNp63* and miR-944, were transfected with the *ΔNp63* promoter-luciferase or the *MIR944* promoter-luciferase construct in combination with the pcDNA (mock vector), ΔNp63α, ΔNp63β or ΔNp63γ expression vectors. After 1 day of incubation, luciferase and β-galactosidase activities were measured. Luciferase activities were normalized to β-galactosidase activities. Data represent the means ± SD of triplicate biological samples and are representative of three different experiments. *P < 0.05 versus pcDNA, unpaired Student's *t*-test. (**E**) Keratinocytes were transfected with 50 nM siRNA against *TP63* mRNA (si-p63) or si-NC. After 1 day, the keratinocytes were transfected with the *MIR944* promoter-luciferase construct. After 2 days of incubation, keratinocytes were lysed, and the luciferase and β-galactosidase activities were measured. Luciferase activities were normalized to β-galactosidase activities. Data represent the means ± SD of triplicate biological samples and are representative of three different experiments. *P < 0.05 versus si-NC, unpaired Student's *t*-test. (**F**) UCSC Genome Browser view of the PhyloP score and the nucleotide alignment of multiple species for the predicted p63-binding sites on the *MIR944* promoter region. In the bottom, the sequences of the wild type and the mutant type were shown. (**G**) Each of three predicted p63-binding sites was mutated by site-directed mutagenesis. WM266-4 melanoma cells were transfected with a wild type or mutant type *MIR944* promoter-luciferase construct in combination with a pcDNA (mock vector), ΔNp63α, or ΔNp63β expression vector. After 1 day of incubation, the luciferase and β-galactosidase activities were measured. Luciferase activities were normalized to β-galactosidase activities. Data represent the means ± SD of triplicate biological samples and are representative of three different experiments. *P < 0.05 versus pcDNA, unpaired Student's *t*-test.

As we had shown that *MIR944* contains its own promoter, it was plausible that miR-944 is generated from a separate transcript and not from the host gene *TP63* transcript. However, as illustrated in Figure 1C, its keratinocyte-specific expression among cell lines, which is concordant with that of *ΔNp63* mRNA expression, suggesting that miR-944 expression is somehow dependent on ΔNp63, requires explanation. One possible explanation is that *MIR944* is the transcriptional target of the ΔNp63 protein. Interestingly, three consensus p63-binding sites were identified on the genomic sequences of the *MIR944* promoter (Figure 3C). Moreover, analysis of the recent ChIP-Seq study of genome-wide p63-binding in human keratinocytes indicated that p63-binding was enriched at the predicted #3 binding site of the *MIR944* promoter region, implying the ΔNp63-mediated activation of this promoter (Figure 4A) (32).

To further confirm this observation, we performed several experiments. Pan-p63 knockdown diminished ΔNp63-binding to the *MIR944* promoter (Figure 4B) and expression of mature miR-944 (Figure 4C). Moreover, overexpression of ΔNp63 elicited a marked increase in the activity of *MIR944* promoter-luciferase (Figure 4D). ΔNp63β isoform showed the greatest increase although all isoform of ΔNp63 were effective for the eliciting the activity *MIR944* promoter. In contrast, p63-depletion blocked the differentiation-induced activation of the *MIR944* promoter (Figure 4E). These results indicated that *MIR944* is a target of ΔNp63.

Next, we investigated to which sites ΔNp63 binds. We first analyzed the conservation of predicted sites, as transcription factors generally bind to conserved sites. Multiple alignment analysis showed that the predicted #3 p63-binding site is widely conserved in vertebrates (Figure 4F). We further performed site-directed mutagenesis of each of the predicted binding sites in the *MIR944* promoter vector to identify the ΔNp63-binding sites more precisely, and found that mutation of the #3 binding site markedly blocked the ΔNp63-mediated activation of the *MIR944* promoter (Figure 4G), meaning that ΔNp63 binds to #3 p63-binding site of the *MIR944* promoter.

**Figure 5.**
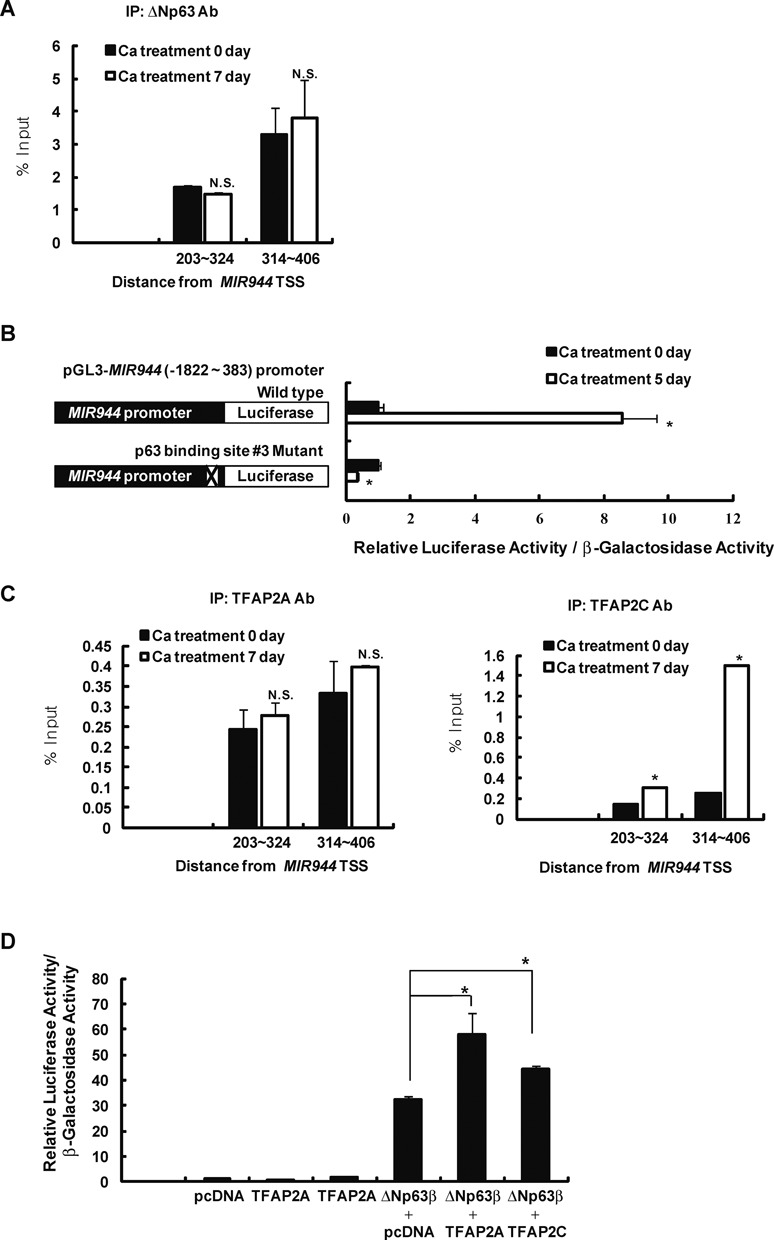

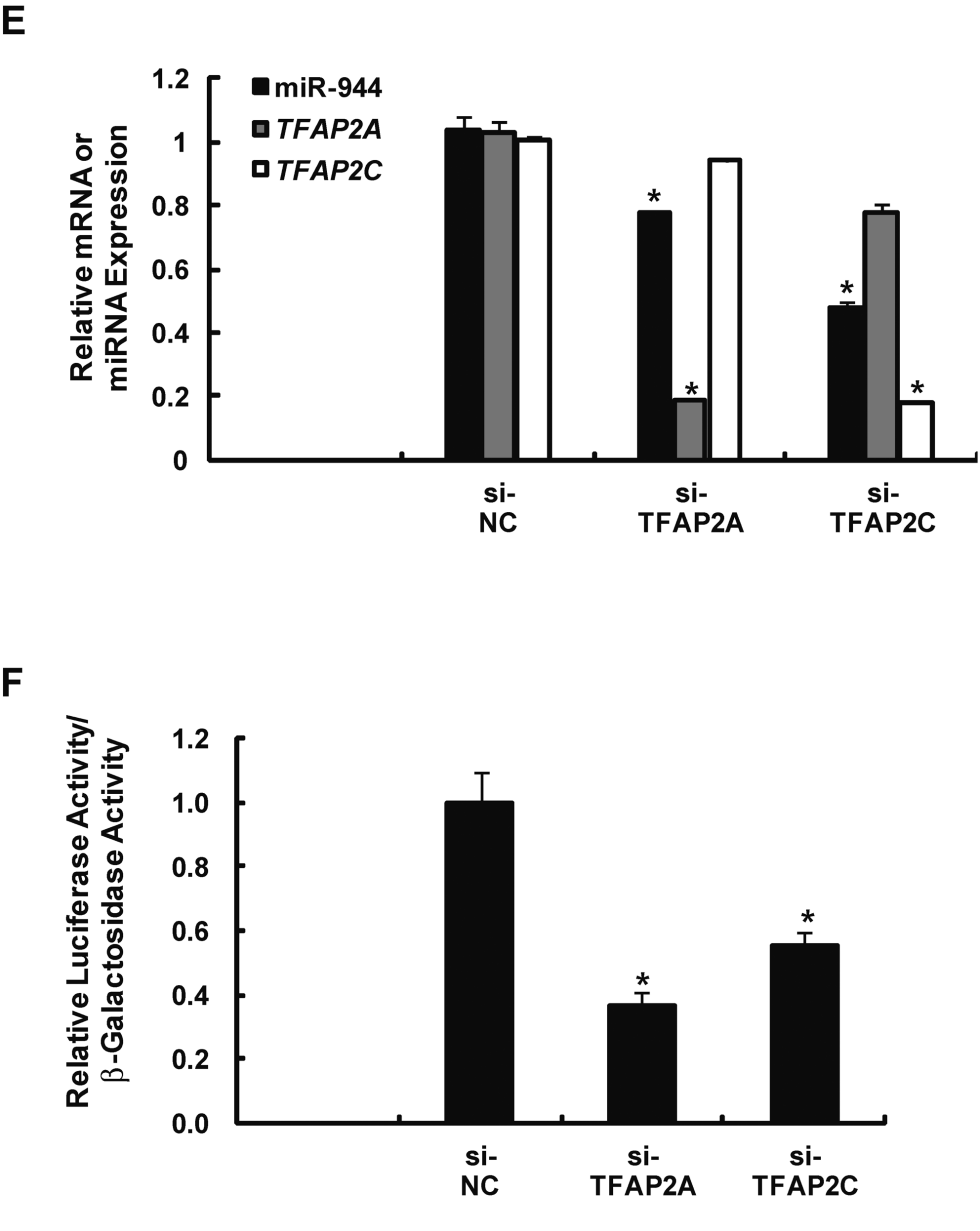
TFAP2A and TFAP2C functionally support the ΔNp63-mediated activity of the *MIR944* promoter. (**A**) ChIP-qPCR results for binding of ΔNp63 to the *MIR944* promoter in undifferentiated and differentiated keratinocytes. Data represent the means ± SD of triplicate biological samples and are representative of three different experiments. (**B**) Keratinocytes were transfected with a *MIR944* promoter-luciferase construct or a p63-binding site #3 mutant of the *MIR944* promoter-luciferase construct. After 5 days of incubation in high-calcium medium, differentiated keratinocytes were lysed, and luciferase and β-galactosidase activities were measured. Luciferase activities were normalized to β-galactosidase activities. Data represent the means ± SD of triplicate biological samples and are representative of three different experiments. *P < 0.05 versus Ca treatment 0 day, unpaired Student's *t*-test. (**C**) ChIP-qPCR results for binding of TFAP2A (left panel) and TFAP2C (right panel) to the *MIR944* promoter in undifferentiated and differentiated keratinocytes. Data represent the means ± SD of triplicate biological samples and are representative of three different experiments. *P < 0.05 versus Ca treatment 0 day, unpaired Student's *t*-test. (**D**) The effect of TFAP2A or TFAP2C overexpression on the ΔNp63-mediated activities of the *MIR944* promoter was examined. WM266-4 melanoma cells were transfected with a *MIR944* promoter-luciferase construct along with the indicated expression vectors. After 1 day of incubation, the luciferase and β-galactosidase activities were measured. Luciferase activities were normalized to β-galactosidase activities. Data represent the means ± SD of triplicate biological samples and are representative of three different experiments. *P < 0.05, unpaired Student's *t*-test. (**E**) Reduction in the expression of miR-944 with TFAP2A or TFAP2C depletion in keratinocytes. Keratinocytes were transfected with 50 nM siRNA against *TFAP2A* (si-TFAP2A), *TFAP2C* (si-TFAP2C), or si-NC. After 2 days, RNA was extracted, and expression levels were analyzed using RT-qPCR. *TFAP2A* and *TFAP2C* mRNA levels were normalized to *RPLP0* expression, and miR-944 expression was normalized to that of *RNU48* small RNA. Data represent the means ± SD of triplicate biological samples and are representative of three different experiments. *P < 0.05 versus si-NC, unpaired Student's *t*-test. (**F**) Keratinocytes were transfected with 50 nM siRNA against *TFAP2A* (si-TFAP2A), *TFAP2C* (si-TFAP2C), or si-NC. The following day, keratinocytes were transfected with a *MIR944* promoter-luciferase construct. After 2 days of incubation, keratinocytes were lysed, and the luciferase and β-galactosidase activities were measured. Luciferase activities were normalized to β-galactosidase activities. Data represent the means ± SD of triplicate biological samples and are representative of three different experiments. *P < 0.05 versus si-NC, unpaired Student's *t*-test.

ΔNp63 expression is gradually reduced in the epidermis during differentiation. However, miR-944 expression is not decreased, even though it is the target of ΔNp63. This raised the question of whether miR-944 biogenesis is sustained during differentiation. Interestingly, we found that ΔNp63-binding to the *MIR944* promoter is maintained during the differentiation of keratinocytes (Figure 5A); additionally, mutation of the ΔNp63-binding site blocked the differentiation-induced increase in the activation of the *MIR944* promoter (Figure 5B).

Thus, we proposed that co-regulators may function to support the binding of ΔNp63 to the *MIR944* promoter, resulting in the maintenance of miR-944 expression. It has recently been reported that AP-2alpha (TFAP2A) and AP-2gamma (TFAP2C) co-operate with p63 at p63-binding sites, resulting in the modulation of p63 transcriptional activity (32). Therefore, we hypothesized that TFAP2A and TFAP2C could be co-regulators of the transcriptional targeting of ΔNp63 to the *MIR944* promoter during epidermal differentiation. First, we examined whether TFAP2A and TFAP2C bind to the *MIR944* promoter during keratinocyte differentiation. ChIP-qPCR with anti-TFAP2A and anti-TFAP2C antibodies showed that TFAP2A bound to the *MIR944* promoter during the differentiation and that TFAP2C enrichment was increased in differentiated keratinocytes (Figure 5C). TFAP2A or TFAP2C overexpression also enhanced the ΔNp63-mediated activation of the *MIR944* promoter, as for other p63 target genes, as described in an earlier report (Figure 5D) (32). Moreover, TFAP2A and TFAP2C depletion reduced the expression of mature miR-944 and the differentiation-induced activation of the *MIR944* promoter (Figure 5E and F). Taken together, these results indicated that miR-944 expression is transcriptionally maintained during epidermal differentiation by the complex interplay between ΔNp63 and AP-2 in the promoter region of this gene.

### miR-944 regulates differentiation in the epidermis

**Figure 6.**
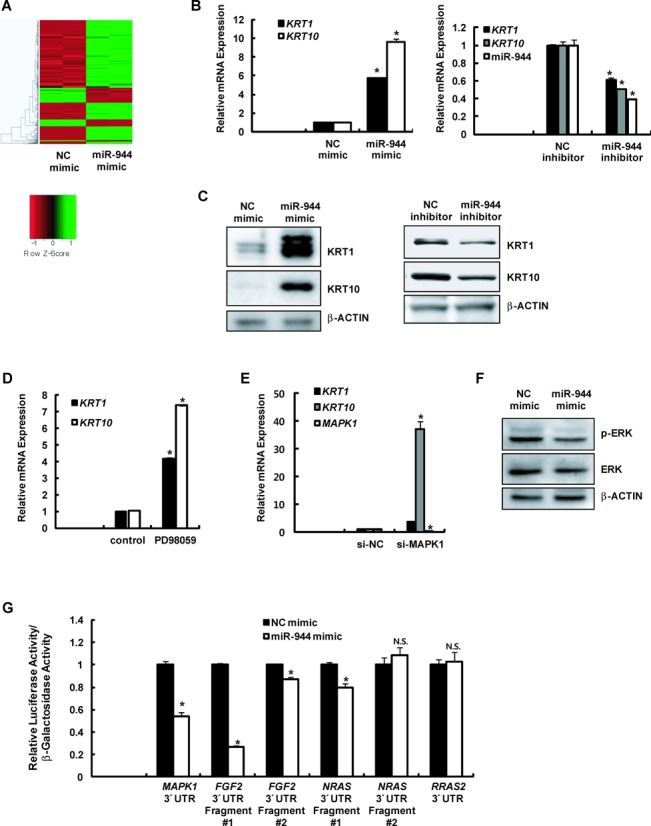

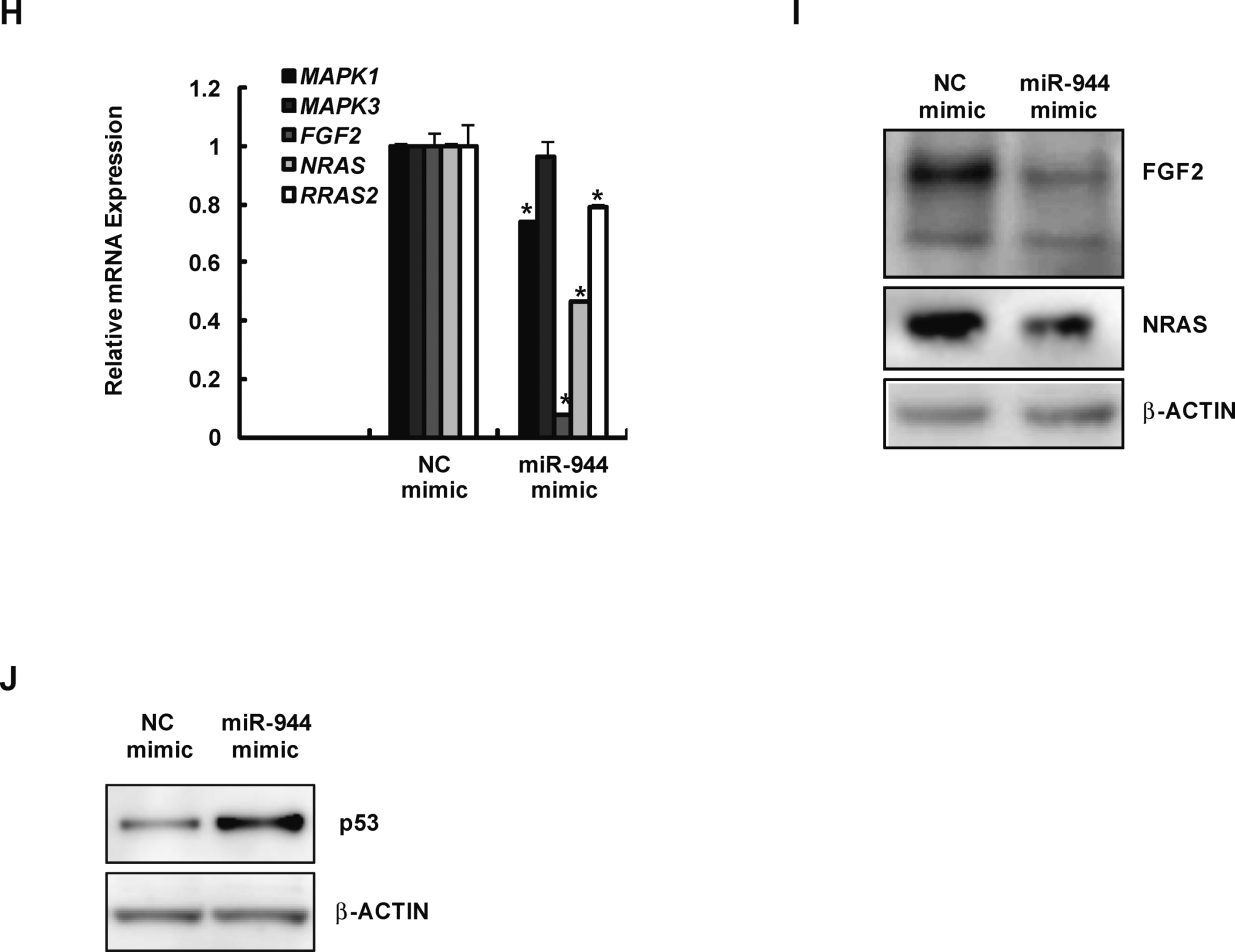
miR-944 modulates keratinocyte differentiation. (**A**) Heat maps of the mRNAs differentially expressed between negative control mimic (NC mimic)- and miR-944 mimic-transfected keratinocytes. (**B**) The mRNA expression levels of early differentiation markers (*KRT1* and *KRT10*) in keratinocytes that had been transfected with 20 nM miR-944 mimic or NC mimic (left panel) or with 50 nM miR-944 inhibitor or negative control inhibitor (NC inhibitor; right panel) were analyzed using RT-qPCR. Expression levels were normalized to *RPLP0* mRNA expression. Data represent the means ± SD of triplicate biological samples and are representative of three different experiments. *P < 0.05 versus NC mimic or NC inhibitor, unpaired Student's *t*-test. (**C**) The protein expression levels of KRT1, KRT10, and β-actin were examined through western blotting after keratinocytes had been transfected with a miR-944 mimic (left panel) or a miR-944 inhibitor (right panel). (**D**) The mRNA expression levels of *KRT1* and *KRT10* in keratinocytes that had been treated with PD98049 for 2 days were analyzed using RT-qPCR. Expression levels were normalized to those of *RPLP0* mRNA. Data represent the means ± SD of triplicate biological samples and are representative of three different experiments. *P < 0.05 versus control, unpaired Student's *t*-test. (**E**) Keratinocytes were transfected with 50 nM siRNA against *MAPK1* mRNA (si-MAPK1) or negative control siRNA (si-NC). After 2 days of incubation, RNAs were extracted, and the expression levels of the indicated genes were analyzed using quantitative real-time PCR (RT-qPCR). Data represent the means ± SD of triplicate biological samples and are representative of three different experiments. *P < 0.05 versus si-NC, unpaired Student's *t*-test. (**F**) The protein expression levels of phospho-ERK, ERK, and β-actin were examined using western blotting after keratinocytes were transfected with a miR-944 mimic. (**G**) The *MAPK1, FGF2, NRAS*, and *RRAS2* 3’-UTR reporter vector was transfected into WM266-4 melanoma cells that had been previously transfected with a miR-944 mimic. The luciferase and β-galactosidase activities were measured after 24 h. The luciferase activities were normalized to the β-galactosidase activities. Data represent the means ± SD of triplicate biological samples and are representative of three different experiments. *P < 0.05 versus NC mimic, unpaired Student's *t*-test. (**H**) The mRNA expression levels of *MAPK1, FGF2, NRAS*, and *RRAS2* in keratinocytes that had been transfected with 20 nM miR-944 mimic or NC mimic (left panel) were analyzed using RT-qPCR. Expression levels were normalized to *RPLP0* mRNA expression. Data represent the means ± SD of triplicate biological samples and are representative of three different experiments. *P < 0.05 versus NC mimic, unpaired Student's *t*-test. (**I**) The protein expression levels of FGF2, NRAS, and β-actin were examined using western blotting after keratinocytes were transfected with a miR-944 mimic. (**J**) The protein expression levels of p53, and β-actin were examined using western blotting after keratinocytes were transfected with a miR-944 mimic.

To characterize the biological function of miR-944 in the epidermis, we investigated genes that were differentially regulated by miR-944 in keratinocytes. Human primary keratinocytes were transfected with a miR-944 mimic, and their gene expression profiles were analyzed on an Affymetrix GeneChip (Figure 6A, and Supplementary Table S4). Interestingly, we found that the expression levels of genes encoding the early differentiation markers keratin 1 (*KRT1*) and keratin 10 (*KRT10*) were markedly increased by miR-944; this finding was confirmed using RT-qPCR (left panel, Figure 6B) and western blotting (left panel, Figure 6C). Furthermore, a miR-944 inhibitor reduced the mRNA and protein expression levels of KRT1 and KRT10 (right panel, Figure 6B; right panel, Figure 6C). However, the mRNA expression levels of late differentiation markers, including filaggrin (*FLG*), involucrin (*IVL*), loricrin (*LOR*), and transglutaminase (*TGM1*), were not significantly altered (Supplementary Figure S8). These results revealed that miR-944 plays a role in regulating the early steps of epidermal differentiation by promoting the expression of early differentiation markers.

Early differentiation of keratinocytes by miR-944 is similar to the effect induced by the inhibition of ERK signaling. Several reports have demonstrated that the suppression of ERK signaling promotes epidermal differentiation (33–35), as our study also confirmed. Treatment with an inhibitor of MAPK signaling (Figure 6D) or transfection with siRNA against MAPK1 (si-MAPK1; Figure 6E) in keratinocytes induced the expression of *KRT1* and *KRT10* mRNAs. Interestingly, we found that MAPK signaling is inhibited by miR-944. Overexpression of miR-944 significantly reduced the expression of phosphorylated ERK and slightly decreased the levels of non-phosphorylated ERK (Figure 6F).

**Figure 7.**
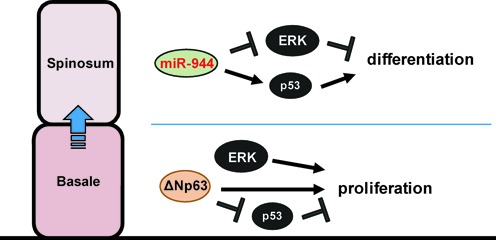
Schematic model of miR-944 biogenesis and function. At the basal layer, ΔNp63 maintains the proliferation of keratinocytes. Moreover, highly active ERK signaling also play a role in proliferation. However, at the spinous layer, ΔNp63 expression is reduced. ΔNp63-induced miR-944 induces cell differentiation. miR-944 functions to reduce ERK signaling and to upregulate p53 expression. These models could explain how ΔNp63 functions in inducing differentiation despite its capacity to maintain proliferation.

Next, we investigated whether miR-944 directly targets genes in the ERK signaling pathway. Interestingly, several miRNA targeting methods predicted that *MAPK1* mRNA could be an important target of miR-944. Moreover, microarray data and the miRNA targeting method using TargetScan algorithm (http://www.targetscan.org/) revealed that miR-944 could also directly target *FGF2*, *NRAS* and *RRAS2* which are involved in the ERK signaling pathway, reducing their mRNA expression. To verify that *FGF2*, *NRAS* and *RRAS2* are the direct targets of miR-944, we performed a 3’UTR reporter-binding assay. The partial regions of 3′UTR that included predicted miR-944 binding sites in *MAPK1, FGF2, NRAS* and *RRAS2* mRNAs were cloned downstream of the luciferase gene in a reporter plasmid. These clones were transfected into cells with the miR-944 mimic or NC mimic and their activities were measured. As shown in Figure 6G, overexpression of miR-944 significantly decreased the 3′UTR activity of *MAPK1* and *FGF2* and slightly reduced that of *NRAS*, but had no effect on the 3′UTR of *RRAS2*. However, the reduction in activities by miR-944 were not observed with mutant type of *MAPK1, FGF2*, and *NRAS* 3′UTR reporter vector containing the mutations in the predicted miR-944 binding site (Supplementary Figure S9), implying that *MAPK1, FGF2* and *NRAS* mRNA are the direct targets of miR-944. Similar to these data, the mRNA expression of *MAPK1, FGF2* and *NRAS* was decreased (Figure 6H) and their protein levels were also reduced by miR-944 (Figure 6F and I). Therefore, we concluded that miR-944 modulates early epidermal differentiation by directly targeting ERK signaling. In addition, it is also known that p53 play a role in the differentiation of keratinocytes (36–38) and we observed that the protein level of p53 was increased by the transfection of miR-944 mimic in keratinocytes (Figure 6J). Therefore, we concluded that miR-944 modulates early epidermal differentiation by directly targeting ERK signaling and upregulating the expression of p53 (Figure 7).

## DISCUSSION

Several intronic miRNAs show discordant expression with their respective host genes. Many models have been proposed to explain this discrepancy, as the biogenesis of intronic miRNAs is complicated and diversified. One feasible explanation is that some intronic miRNAs have their own promoters and are produced from unique transcription units that are distinct from those of the host-gene precursor mRNAs. Although bioinformatics analysis has demonstrated that >30% of intronic miRNAs may be generated from their own promoters, little is known about the mechanisms involved in the regulation of these promoters. In this study, we demonstrated that *MIR944*, which is located in the intron of *ΔNp63* has its own promoter: an open chromatin region approximately 5 kb downstream of the *MIR944* stem-loop sequence has promoter activity for the transcription of primary *MIR944*. Moreover, interestingly, we revealed that miR-944 expression also depends on ΔNp63 protein at the transcriptional level. The ΔNp63 protein directly binds to the *MIR944* promoter and drives transcription in keratinocytes, forming a unique regulatory mechanism, in which intronic miRNA is generated through the transcriptional activity of its host gene. To our knowledge, this report is the first description of a mechanism regulating intronic miRNA that is both dependent on and independent from its host gene.

Our experimental evidence indicated that miR-944 is generally expressed in cells in which ΔNp63 is specifically expressed, although *MIR944* transcription is distinct from the transcription of its host gene, *ΔNp63*. We solved this dilemma by finding that*MIR944* is a target gene of ΔNp63. However, although the transcriptional activity of ΔNp63 is critical for miR-944 expression in keratinocytes, ΔNp63 overexpression did not lead to production of endogenous miR-944 in WM266-4 melanoma cells (data not shown), indicating that other factors may be required for initiation of transcription. We suggest that sharing of chromatin dynamics within the genomic region of *ΔNp63* is another factor necessary for the initiation of its transcription. In keratinocytes, the region encompassing the *MIR944* promoter has a relatively open chromatin structure, in contrast to the corresponding region in WM266-4 melanoma cells, which do not express *ΔNp63* mRNA and miR-944. The open chromatin architecture of the *ΔNp63* genomic region in keratinocytes may modulate the initiation of transcription of *MIR944*. Furthermore, we identified AP-2 as a co-regulator that reinforces the binding of ΔNp63 to the consensus region of the *MIR944* promoter. In addition to the open chromatin structure, several co-regulators, including AP-2, may play critical roles in *MIR944* transcription and may thus modulate the regulation of miR-944 expression.

*MIR944* is a relatively recently evolved gene. The translational bioinformatics research of Jianjun Chen's group has suggested that evolutionarily non-conserved young miRNAs tend to be less frequently co-expressed with their host genes than are conserved miRNAs. Our findings regarding the mechanism of miR-944 biogenesis is consistent with this suggestion. Additionally, it is known that evolutionarily young miRNAs are preferentially expressed in specific tissues (27,39). Consistent with this notion, miR-944 is specifically expressed in JAR placental cells and keratinocytes (Figure 1C). Moreover, miR-944-expressing cells express *ΔNp63* mRNA, but not *TAp63* mRNA (Figure 1C). This observation may indicate that the *MIR944* transcript differs from that of its host gene, because if miR-944 were also generated from the pre-mRNA of the host gene, its expression would also be detected in TAp63-expressing cells. Therefore, we suggest that the promoter region of miR-944 is active only in *ΔNp63* mRNA-expressing cells. Further research into the three-dimensional chromatin complex may help to elucidate the exact mechanism of miR-944 biogenesis.

It is well known that ΔNp63 is crucial for epithelial development. A loss-of-function study using knockout mice showed that ΔNp63-deficiency resulted in defects in epidermis formation (40). ΔNp63 expression is highest in the basal layer of the epidermis, where it maintains the proliferative capability of cells through the MYC-regulated gene network and the regulation of the Skp2-p130 level and by suppressing anti-proliferative genes related to p53 signaling (6,41). In contrast, previous studies have established that ΔNp63 in the epidermis plays a role in the commitment of keratinocytes to differentiation. The depletion of ΔNp63 results in a defect in the expression of early differentiation markers in organotypic culture and *in vitro* (5,6). Furthermore, ΔNp63 regulates the cell adhesion-related gene network, including genes encoding fibronectin 1, interleukin-1, and jagged-1, which are involved in keratinocyte differentiation (6). However, the molecular mechanism underlying induction of epidermal differentiation by ΔNp63 and how ΔNp63 could play dual roles in proliferation and differentiation of keratinocytes has not been investigated in depth.

Here, we suggest that *MIR944*, a ΔNp63 target gene, could at least partially explain this matter. As illustrated in our results, the promoter region of *MIR944* is active only in *ΔNp63* mRNA-expressing cells, but *MIR944* promoter activity is not linearly dependent on the quantity of ΔNp63. Most likely, miR-944 expression is transcriptionally maintained during epidermal differentiation due to reinforcement of the binding of ΔNp63 to the consensus region of the *MIR944* promoter by the assistance of its co-regulator AP-2. Our results also showed that miR-944 modulates the expression of the early differentiation markers KRT1 and KRT10, indicating that miR-944 is involved in ΔNp63 network that regulates the differentiation of keratinocytes. Therefore, taken together, our results infer that at the basal layer, this differentiation-driving potential of miR-944 may be neutralized by the opposing action of ΔNp63 and its downstream signaling. However, at the spinous layer or under any differentiation-initiation stress, expression of ΔNp63 is decreased, but is still sufficient to maintain the expression of miR-944 due to the unique mechanism for regulating its expression. When the regulatory effect of ΔNp63 on cell proliferation weakens, miR-944 induces the onset of early differentiation. Interestingly, we demonstrated that miR-944 inhibits ERK signaling. It is known that ERK signaling is activated at the basal layer and plays a role in the proliferation of keratinocytes (34,42). Moreover, we also found that p53 expression is increased by miR-944 overexpression (Figure 6J). Furthermore, it is known that p53 activity is increased after ΔNp63 is withdrawn during epidermal differentiation (36) and is upregulated in differentiated epidermis (37). Additionally, the knock-down of p53 reduced the KRT1 synthesis in human primary neonatal foreskin keratinocytes (38), meaning that p53 plays an important role in the differentiation of keratinocytes. These findings suggest that miR-944 contributes to the early differentiation of keratinocytes after these cells lose their proliferation potential.

Overall, our study demonstrates that miR-944, which is histologically specific to keratinocytes and is evolutionarily specific to primates, is generated from its own transcript via the interplay of ΔNp63 and AP2. Moreover, miR-944, as a direct target of ΔNp63, is involved in the ΔNp63 gene network responsible for the onset of epidermal differentiation, by regulating ERK and p53 signaling.

## SUPPLEMENTARY DATA

Supplementary Data are available at NAR Online.

SUPPLEMENTARY DATA
