# Supplementary material for: ΔNp63 intronic miR-944 is implicated in the ΔNp63-mediated induction of epidermal differentiation
Source: Nucleic Acids Res. 2015 Jul 21;43(15):7462–79. doi: 10.1093/nar/gkv735 (PMC4551945; doi:10.1093/nar/gkv735)
Supplement: SUPPLEMENTARY DATA [file supp_43_15_7462__index.html]

ΔNp63 intronic miR-944 is implicated in the ΔNp63-mediated induction of epidermal differentiation — ΔNp63 intronic miR-944 is implicated in the ΔNp63-mediated induction of epidermal differentiation — SUPPLEMENTARY DATA 

# ΔNp63 intronic miR-944 is implicated in the ΔNp63-mediated induction of epidermal differentiation

## SUPPLEMENTARY DATA

- SUPPLEMENTARY DATA
- SUPPLEMENTARY DATA
- SUPPLEMENTARY DATA
- SUPPLEMENTARY DATA
- SUPPLEMENTARY DATA
