# Supplementary material for: ΔNp63 intronic miR-944 is implicated in the ΔNp63-mediated induction of epidermal differentiation
Source: Nucleic Acids Res. 2015 Jul 21;43(15):7462–79. doi: 10.1093/nar/gkv735 (PMC4551945; doi:10.1093/nar/gkv735)
Supplement: SUPPLEMENTARY DATA [file supp_gkv735_nar-03662-a-2014-File012.pdf]

**Supplemental Figure S1.**

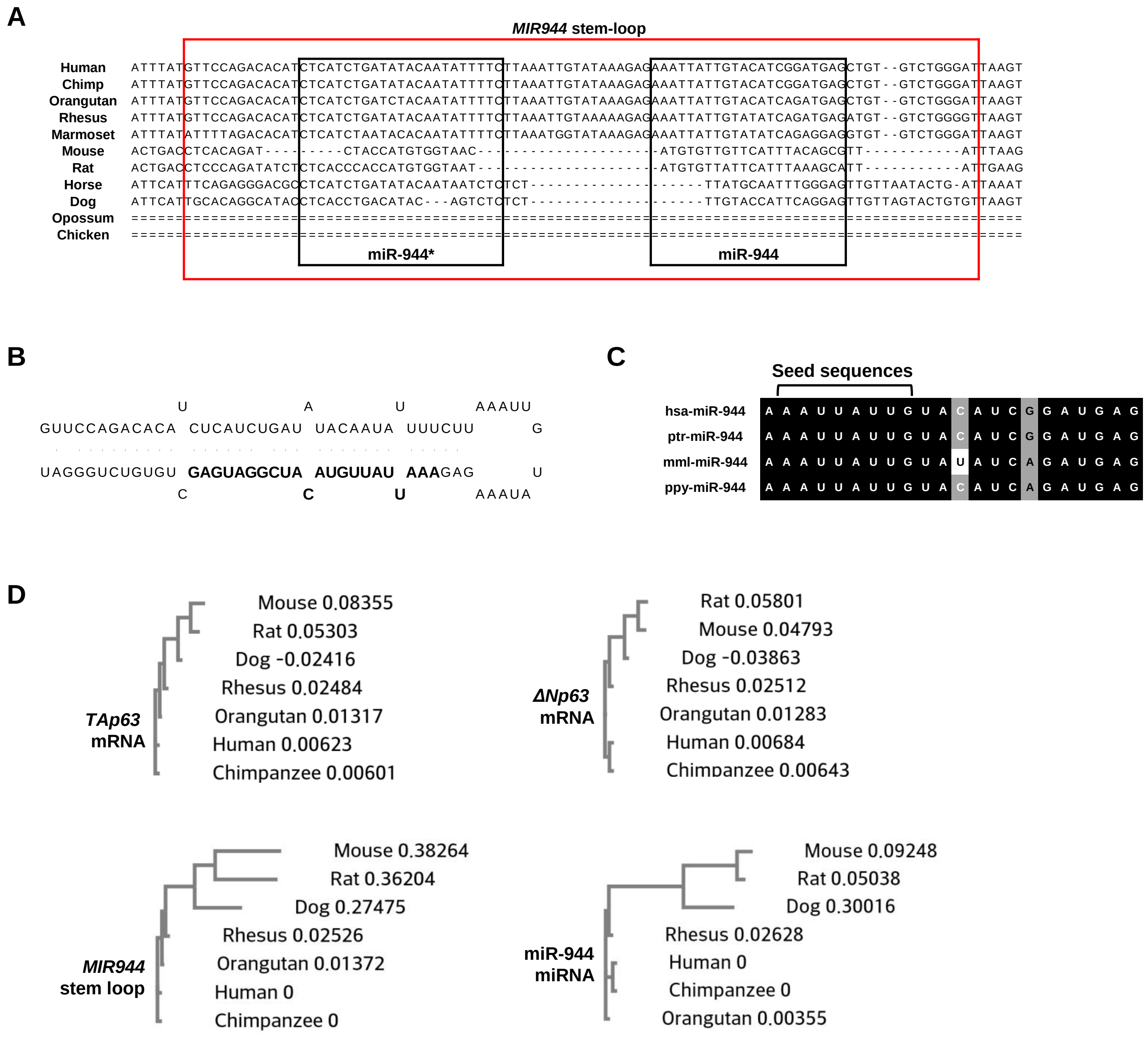


**Figure S1. Genomic locations of *MIR944***

(A) Sequence alignment of the *MIR944* stem loop in vertebrates. The miR-944 and miR-944* are outlined in black boxes, and the *MIR944* stem loops are outlined in the red box.

(B) Stem loop structure of duplex of the *mir-MIR944*. Bold letters indicate the sequence of human miR-944.

(C) Sequence alignment of the mature miR-944s of 4 primates from the miRBase Sequence Database (http://www.mirbase.org/). Black and grey boxes indicate sequence identity or similarity, respectively. Predicted seed sequences are indicated (hsa, Homo sapiens; ptr, Pan troglodytes; mml, Macaca mulatta; ppy, Pongo pygmaeus).

(D) Phylogenetic tree of *TAp63* mRNA, Δ*Np63* mRNA, *MIR944* and mature miR-944 in mammals. Bioinformatics analysis was performed using the Homologene database (http://www.ncbi.nlm.nih.gov/homologene) and Clustal Omega (https://www.ebi.ac.uk/Tools/msa/clustalo/).

**Supplemental Figure S2.**

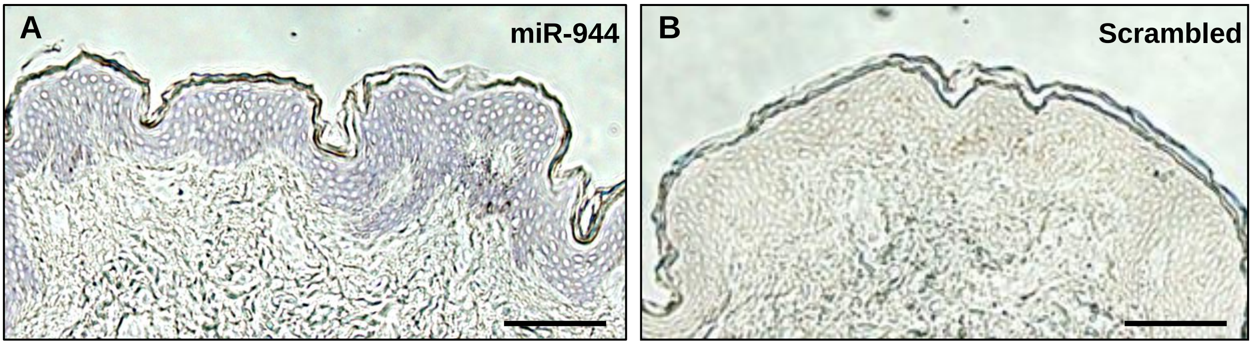


**Figure S2. Detection of miR-944 in the epidermis of human skin**

In situ hybridization was performed on normal human skin sections using either an miR-944-specific LNA probe (A) or a scrambled probe (B). Bar = 5 μM.

**Supplemental Figure S3.**

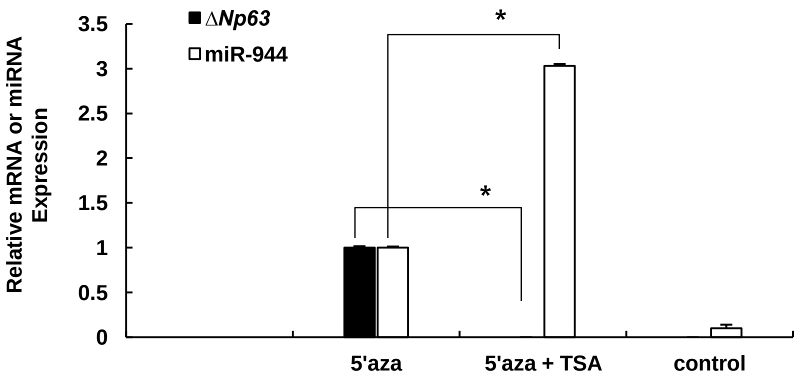


**Figure S3. Independent gene regulation of Δ*Np63* and miR-944 expression in HeLa cells**
The relative Δ*Np63*, and miR-944 expression levels in response to epigenetic modifying agents were quantified. HeLa cells were treated with 5 μM 2′-deoxy-5-azacytidine (5′aza) for 3 days. Subsequently, 1 μM trichostatin A (TSA) was added directly to the medium. After incubating for 1 more day, RNA was extracted, and RT-qPCR analysis was performed. The values obtained from samples treated with only 5′aza are set as 1. Data represent the means ± SD of triplicate biological samples and are representative of three different experiments. *$P < 0.05$, unpaired Student's *t*-test.

**Supplemental Figure S4.**

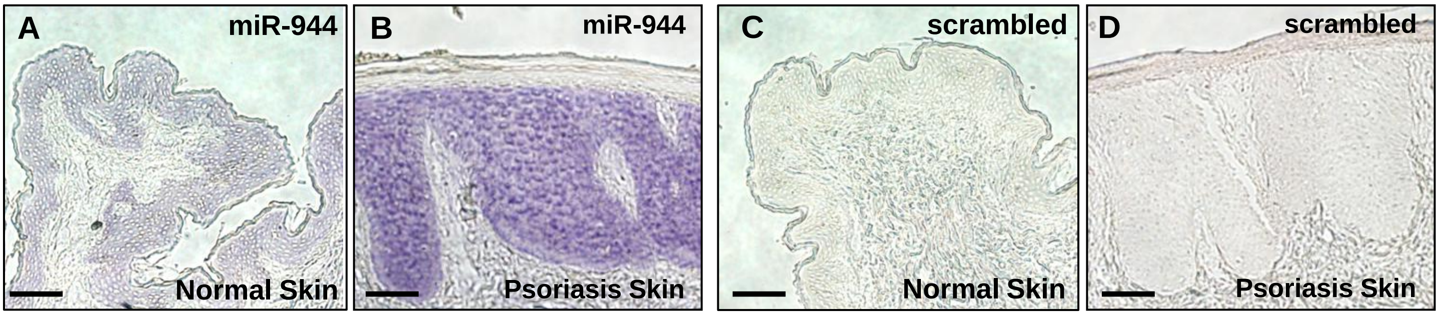


**Figure S4. Detection of miR-944 in normal and psoriatic skin**
In situ hybridization was performed on sections of normal skin (A and C) and psoriatic skin (B and D) using either an miR-944-specific LNA probe (A and B) or a scrambled probe (C and D). Bar = 5 μM.

**Supplemental Figure S5.**

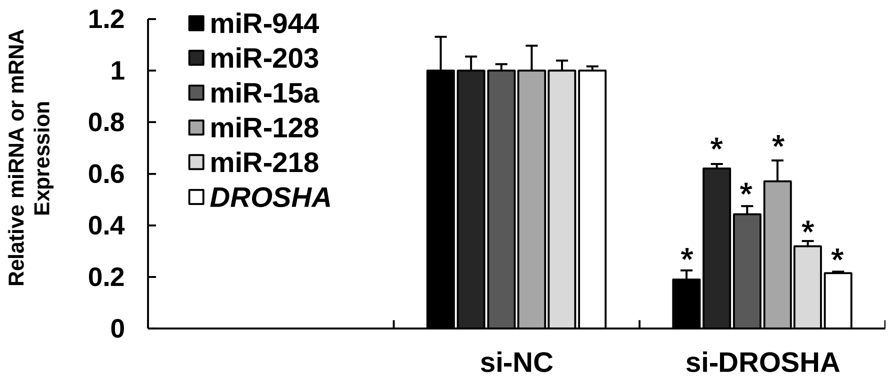


**Figure S5. Significant reduction of miR-944 in *DROSHA*-depleted HaCaT cells**
HaCaT cells were transfected with 50 nM siRNA against *DROSHA* mRNA (si-DROSHA) or negative control siRNA (si-NC). After 2 days of incubation, RNAs were extracted, and the expression levels of the indicated genes were analyzed using quantitative real-time PCR (RT-qPCR). *DROSHA* expression was normalized to ribosomal protein large P0 (*RPLP0*) expression, and miRNA expression was normalized to *RNU48* small RNA expression. Data represent the means ± SD of triplicate biological samples and are representative of three different experiments. *P < 0.05 versus si-NC, unpaired Student's *t*-test.

**Supplemental Figure S6.**

A

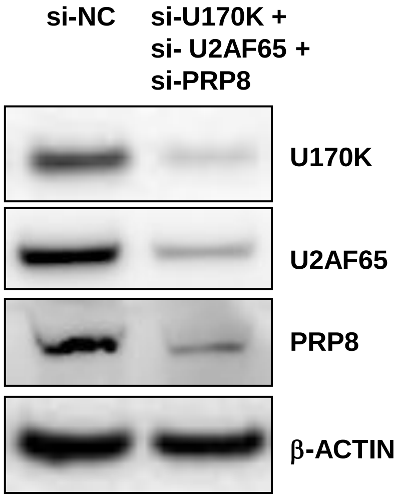


B

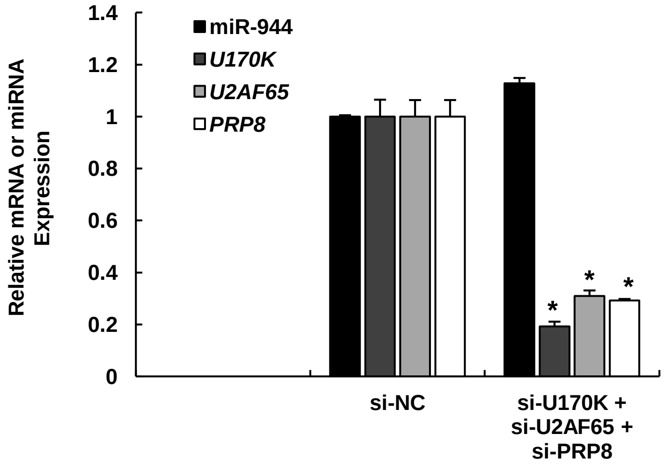


**Figure S6. Splicing factors do not affect miR-944 biogenesis**

(A) Three different core components of the spliceosome, U170K, PRP8 and U2AF65 were depleted in human primary keratinocytes by transfecting the cells with the respective siRNAs. After 3 days, proteins were extracted, and the expression levels were analyzed using western blot.

(B) After 3 days, RNAs were extracted, and the expression levels were analyzed using RT-qPCR. The mRNA of *U170K*, *U2AF65* and *PRP8* expression levels were normalized to *RPLP0* mRNA expression, and miR-944 expression was normalized to *RNU48* small RNA expression. Data represent the means ± SD of triplicate biological samples and are representative of three different experiments. *$P < 0.05$ versus , unpaired Student's *t*-test.

## Supplemental Figure S7.

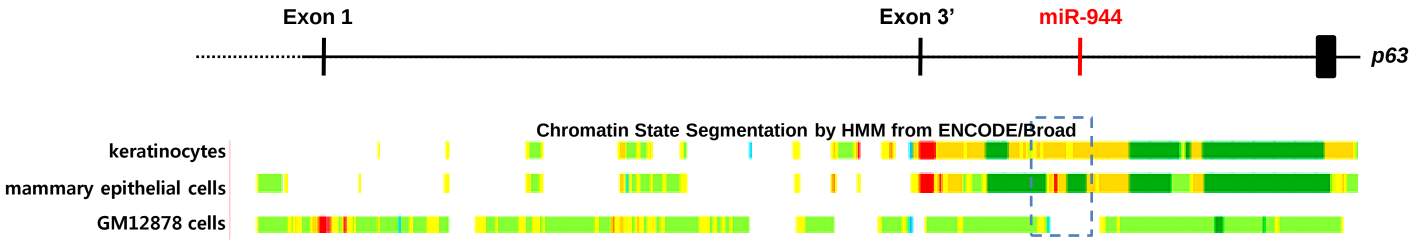


**Figure S7. Prediction of the host gene-independent promoter of *MIR944* transcription**
The UCSC Genome Browser view of the chromatin state segmentation tracks obtained for keratinocytes, mammary epithelial cells, and GM12878 cells at the *p63* gene. By integrating ChIP-Seq data using a Hidden Markov Model (HMM), the chromatin states were segmented and colored to highlight their predicted functional elements (Red, promoter; Yellow, weak and poised enhancer; Green, transcriptional transition). The predicted promoter for *MIR944* is shown in a blue box (dashed line).

**Supplemental Figure S8.**

A

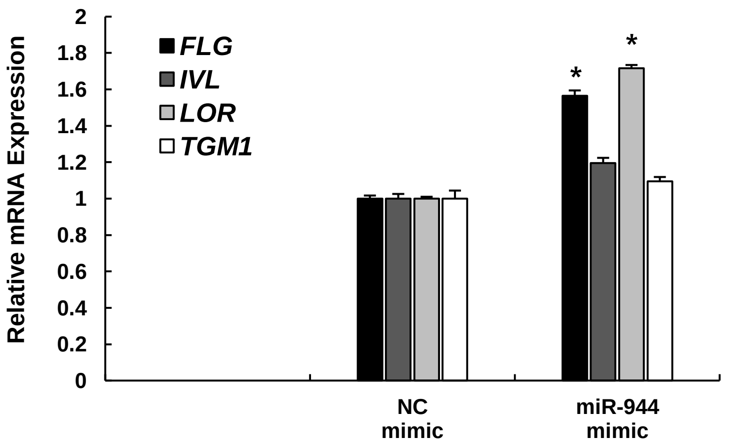


B

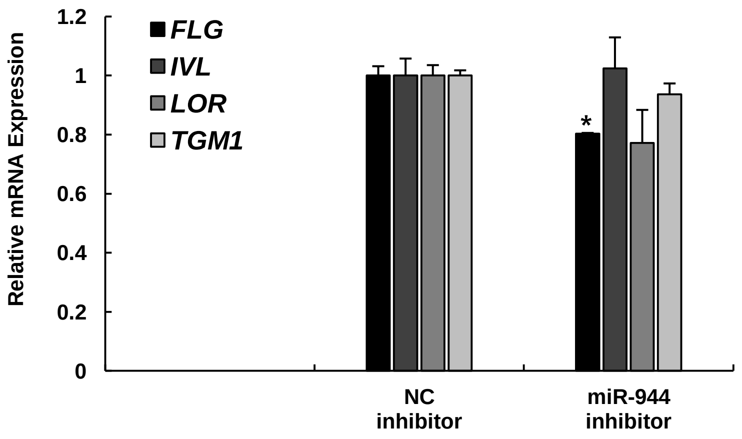


**Figure S8. miR-944 did not affect the expression of late differentiation markers**

The mRNA expression levels of late differentiation markers (*FLG*, *IVL*, *LOR* and *TGM1*) in keratinocytes transfected with 20 nM miR-944 mimic or NC mimic (A) or with 50 nM miR-944 inhibitor or NC inhibitor (B) were analyzed using RT-qPCR. Each expression level was normalized to *RPLP0* mRNA expression. Data represent the means ± SD of triplicate biological samples and are representative of three different experiments. *P < 0.05 versus NC mimic or NC inhibitor, unpaired Student's *t*-test.

**Supplemental Figure S9.**

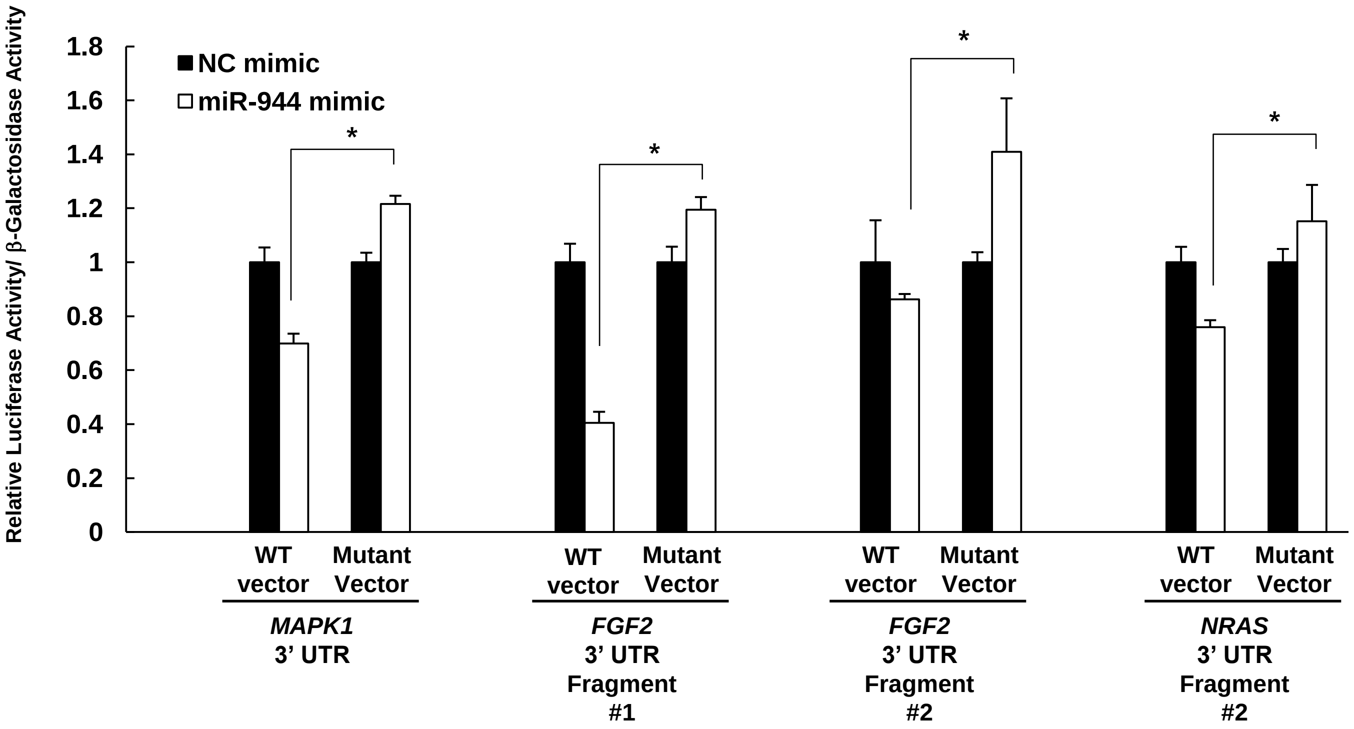


**Figure S9. miR-944 targets the 3′UTR of *MAPK1, FGF2,* and *NRAS* mRNA**

The wild type or mutant type of *MAPK1*, *FGF2*, *NRAS*, and 3'-UTR reporter vector was transfected into WM266-4 melanoma cells that had been previously transfected with a miR-944 mimic. The luciferase and β-galactosidase activities were measured after 24 h. The luciferase activities were normalized to the β-galactosidase activities. Data represent the means ± SD of triplicate biological samples and are representative of three different experiments. *$P$ <0.05 , unpaired Student's $t$-test.
